# Heterogeneity of Glycolytic Phenotype Determined by ^18^F-FDG PET/CT Using Coefficient of Variation in Patients with Advanced Non-Small Cell Lung Cancer

**DOI:** 10.3390/diagnostics13142448

**Published:** 2023-07-22

**Authors:** Sara Pellegrino, Rosa Fonti, Armin Hakkak Moghadam Torbati, Roberto Bologna, Rocco Morra, Vincenzo Damiano, Elide Matano, Sabino De Placido, Silvana Del Vecchio

**Affiliations:** 1 Department of Advanced Biomedical Sciences, University of Naples “Federico II”, 80131 Naples, Italy; sara.pellegrino@unina.it (S.P.); rosa.fonti@unina.it (R.F.); hakakarmin@gmail.com (A.H.M.T.); robertobologna@hotmail.it (R.B.); 2 Department of Clinical Medicine and Surgery, University of Naples “Federico II”, 80131 Naples, Italy; rocco.mor4@gmail.com (R.M.); vincenzo.damiano@unina.it (V.D.); ematano@unina.it (E.M.); deplacid@unina.it (S.D.P.)

**Keywords:** Coefficient of Variation, ^18^F-FDG PET/CT, heterogeneity, Non-Small Cell Lung Cancer, Metabolic Tumor Volume, prognosis

## Abstract

We investigated the role of Coefficient of Variation (CoV), a first-order texture parameter derived from ^18^F-FDG PET/CT, in the prognosis of Non-Small Cell Lung Cancer (NSCLC) patients. Eighty-four patients with advanced NSCLC who underwent ^18^F-FDG PET/CT before therapy were retrospectively studied. SUVmax, SUVmean, CoV, total Metabolic Tumor Volume (MTV_TOT_) and whole-body Total Lesion Glycolysis (TLG_WB_) were determined by an automated contouring program (SUV threshold at 2.5). We analyzed 194 lesions: primary tumors (*n* = 84), regional (*n* = 48) and non-regional (*n* = 17) lymph nodes and metastases in liver (*n* = 9), bone (*n* = 23) and other sites (*n* = 13); average CoVs were 0.36 ± 0.13, 0.36 ± 0.14, 0.42 ± 0.18, 0.30 ± 0.14, 0.37 ± 0.17, 0.34 ± 0.13, respectively. No significant differences were found between the CoV values among the different lesion categories. Survival analysis included age, gender, histology, stage, MTV_TOT_, TLG_WB_ and imaging parameters derived from primary tumors. At univariate analysis, CoV (*p* = 0.0184), MTV_TOT_ (*p* = 0.0050), TLG_WB_ (*p* = 0.0108) and stage (*p* = 0.0041) predicted Overall Survival (OS). At multivariate analysis, age, CoV, MTV_TOT_ and stage were retained in the model (*p* = 0.0001). Patients with CoV > 0.38 had significantly better OS than those with CoV ≤ 0.38 (*p* = 0.0143). Patients with MTV_TOT_ ≤ 89.5 mL had higher OS than those with MTV_TOT_ > 89.5 mL (*p* = 0.0063). Combining CoV and MTV_TOT_, patients with CoV ≤ 0.38 and MTV_TOT_ > 89.5 mL had the worst prognosis. CoV, by reflecting the heterogeneity of glycolytic phenotype, can predict clinical outcomes in NSCLC patients.

## 1. Introduction

Lung cancer is the leading cause of cancer-related death worldwide [1]. Due to the late onset of clinical symptoms, most patients are already in advanced stages having distant metastases and poor overall survival at diagnosis. Based on their molecular and immunophenotypic profiles, these patients are candidates for chemotherapy, targeted therapy or immunotherapy. However, after an initial good response to therapy, the majority of these patients will become resistant to treatment and develop disease progression or die. Therefore, it would be helpful to identify from the beginning those with a higher risk of disease progression and death allowing the adoption of more aggressive therapeutic regimens. The tumor stage at initial diagnosis is the most reliable prognostic factor in Non-Small Cell Lung Cancer (NSCLC) patients and is used to establish subsequent therapeutic strategies. Nevertheless, patients within the same stage can show a wide spectrum of treatment responses and clinical outcomes highlighting the need for additional prognostic factors for a better stratification of these patients.

Texture analysis is an emerging tool for assessing intratumoral heterogeneity in medical imaging allowing to extract clinically relevant subvisual information from images obtained with different modalities, such as Computed Tomography (CT), 2-[^18^F]fluoro-2-deoxy-D-glucose positron emission/computed tomography (^18^F-FDG PET/CT), Magnetic Resonance Imaging (MRI) [2,3,4,5]. Intratumoral heterogeneity of biological, molecular and pathological traits has been considered the main cause of treatment failure, therapeutic resistance and poor overall survival in cancer patients with metastatic disease [6,7,8]. Therefore, assessing tumor heterogeneity could be extremely useful to characterize tumor aggressiveness and to select risk-adapted therapy in individual patients. Similarly, among clinical diagnostic images, heterogeneity of ^18^F-FDG uptake within tumors has been attributed to several factors, including cellularity, proliferation, angiogenesis, necrosis and hypoxia [9], and a high ^18^F-FDG uptake has been often associated with more aggressive tumors, poorer response to treatment and worse prognosis [10].

Previous studies performing texture analysis of ^18^F-FDG PET/CT images in lung cancer patients showed that several parameters including dissimilarity, asphericity, coarseness and entropy were able to predict both Progression-Free Survival (PFS) and Overall Survival (OS) of patients [11,12,13,14,15,16]. Although we are aware that texture analysis is a powerful tool to evaluate tumor heterogeneity, we aimed at obtaining an easy and clinically suitable imaging parameter for the characterization of tumor heterogeneity. To this end, we selected Coefficient of Variation (CoV, Standard Deviation divided by SUVmean) as a simple and easy to calculate first-order texture parameter that may reflect the heterogeneity of glycolytic phenotype.

The aim of our study was to test the ability of CoV derived from ^18^F-FDG PET/CT images in the evaluation and the quantification of the heterogeneity of glycolytic phenotype in primary and metastatic lesions of NSCLC patients with advanced stages. Furthermore, we evaluated the prognostic power of this simple parameter determined on primary tumors and its ability to predict OS and PFS along with other PET-based volumetric parameters such as total Metabolic Tumor Volume (MTV_TOT_) and whole-body Total Lesion Glycolysis (TLG_WB_) measured on all tumor lesions in each patient.

## 2. Materials and Methods

### 2.1. Patients

Our study included 84 consecutive patients (59 men, 25 women; mean age 66 ± 12 years; range 38–87 years) with histologically proven non-small cell lung cancer in advanced disease (stages III and IV) who had undergone whole-body ^18^F-FDG PET/CT scan before any therapy at our Institution (Table 1). This retrospective study has been approved by the institutional ethics committee (Protocol N. 352/18) and all subjects signed an informed consent form.

We studied 41 patients with adenocarcinoma, 20 with squamous cell carcinoma, 3 with large cell carcinoma and 20 with NSCLC Not Otherwise Specified (NOS). Twenty-seven patients were in stage III (7 IIIA, 11 IIIB and 9 IIIC) while 57 patients were in stage IV (20 IVA and 37 IVB). Patients were treated according to their stage and other factors such as histology, molecular pathology, age, performance status and comorbidities [17]. In particular, 69 patients underwent chemotherapy, 4 of which in association with radiotherapy and 15 with immunotherapy. The remaining 15 patients did not receive any specific cancer therapy due to advanced age or severe comorbidities.

Patients were then monitored and the mean follow-up period was 11 months (range 1–58 months). PFS was measured from the date of the baseline ^18^F-FDG PET/CT to the first observation of a progressive disease, relapse or death. OS was calculated from the date of the baseline ^18^F-FDG PET/CT to the date of death.

### 2.2. ^18^F-FDG PET/CT Study

^18^F-FDG PET/CT scans were acquired after fasting for 8 h and 60 min after intravenous administration of 370 MBq (10 mCi) of ^18^F-FDG. The blood glucose level, measured just before tracer administration, was <120 mg/dL in all patients. Hybrid imaging was performed with an Ingenuity TF scanner (Philips Healthcare, Best, The Netherlands). A multidetector CT scan was acquired using the following parameters: 120 kV, 80 mAs, 0.8 s rotation time, and pitch of 1.5; a fully diagnostic contrast-enhanced CT was acquired if not previously performed. PET scan was performed in 3-dimensional mode using 3 min per bed position and six to eight-bed positions per patient, depending on patient height. Iterative image reconstruction was performed with an ordered subsets-expectation maximization algorithm. Attenuation-corrected emission data were obtained using filtered back projection of CT reconstructed images (Gaussian filter with 8 mm full-width half maximum) to match the PET resolution. Transaxial, sagittal, and coronal images as well as coregistered images were preliminary examined using Ingenuity TF software (IntelliSpace Portal V5.0).

### 2.3. ^18^F-FDF PET/CT Image Analysis

PET/CT data were transferred in DICOM format to a workstation and processed by the LIFEx program [18]. All areas of focal ^18^F-FDG uptake visible on 2 contiguous PET slices at least and not corresponding to physiological tracer uptake were considered to be positive. In case of multiple regional or non-regional lymph nodes, liver, bone or metastases in other sites the lesion with the highest SUVmax was analyzed for each category. A Volume of Interest (VOI) of each lesion was delineated on PET images by drawing a tridimensional region around the target lesion using an automated contouring program setting an absolute threshold for SUV at 2.5, in agreement with previous studies [19,20]. Areas of necrosis were not included in the region of interest and were carefully excluded from the analysis. In addition, the accuracy of lesion delimitation was confirmed on the corresponding CT images. By computed analysis of each VOI, the following parameters were obtained: SUVmean, CoV, SUVmax, MTV and Total Lesion Glycolysis (TLG). CoV was determined as Standard Deviation (SD) divided by SUVmean whereas MTV_TOT_ and TLG_WB_ were calculated by the sum of the corresponding values for all primary tumors, lymph nodes and distant metastatic lesions of each patient [21]. Multiple coalescent lymph nodes were considered as a single lesion. Brain metastases were not included in the analysis because of the physiological high FDG avidity of the brain that can affect the correct delineation of the regions of interest. Moreover, not measurable disseminated metastases were also excluded.

### 2.4. Statistical Analysis

Statistical analysis was performed using the software MedCalc for Windows, version 10.3.2.0 (MedCalc Software, Mariakerke, Belgium). A probability value of <0.05 was considered statistically significant. Student’s *t*-test was used to compare the means of unpaired data. Pearson’s correlation coefficient was used to evaluate the linear relationship between continuous variables. Univariate and multivariate analyses of clinical and imaging variables were performed using Cox proportional hazards regression. Variables that predicted PFS and OS by univariate analysis were included in the model for multivariate analysis along with age, the latter independently from its statistical significance. Survival analysis was performed using the Kaplan–Meier method and log-rank tests. Survivors were censored at the time of the last clinical control.

## 3. Results

**Figure 1:**
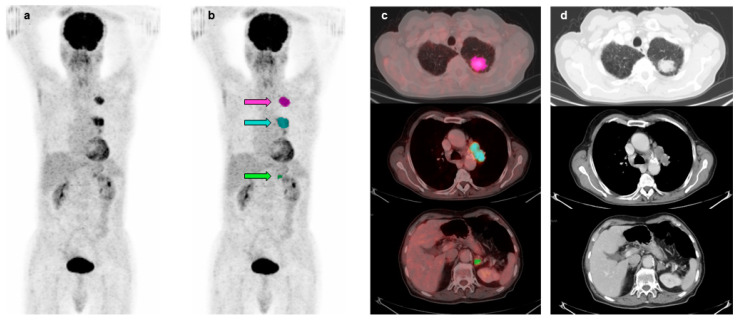
Representative images of ^18^F-FDG PET/CT scan in an 80-year-old patient with stage IVA NSCLC. Maximal intensity projection views without (**a**) and with an overlay of MTVs on primary tumor (pink arrow), lymph node (light blue arrow) and adrenal metastasis (green arrow) (**b**). Transaxial fusion images with overlay of MTVs on primary tumor (pink), lymph node (light blue) and adrenal metastasis (green) (**c**). Corresponding transaxial CT images (**d**). MTV_TOT_ = 42.67 mL.

^18^F-FDG PET/CT scans of 84 patients with advanced NSCLC were studied and conventional and volumetric imaging parameters were obtained. In particular, imaging parameters such as SUVmax, SUVmean, and CoV were derived from primary tumors (mean diameter 4.6 ± 2.6 cm, range 1.2–13 cm) as well as from the metastatic lesions showing the highest SUVmax within each category. In particular, 84 primary lung tumors, 48 regional lymph nodes, 17 non-regional lymph nodes, 9 liver metastases, 23 bone lesions and 13 metastases in other sites were included in the analysis. Figure 1 shows representative images of the VOIs drawn around primary tumor, lymph node and distant metastases in a patient with stage IVA NSCLC.

Mean SUVmax, SUVmean and CoV values were 12.17 ± 5.86, 5.44 ± 2.04 and 0.36 ± 0.13 in primary tumors, 10.97 ± 6.96, 4.67 ± 1.85 and 0.36 ± 0.14 in regional lymph nodes, 14.22 ± 10.41, 5.40 ± 2.11 and 0.42 ± 0.18 in non-regional lymph nodes, 9.90 ± 4.67, 5.12 ± 1.48 and 0.30 ± 0.14 in liver metastases, 10.68 ± 5.21, 4.46 ± 1.00 and 0.37 ± 0.17 in bone lesions and 10.36 ± 3.82, 4.79 ± 1.19 and 0.34 ± 0.13 in other distant metastases (Table 2).

No statistically significant differences were found between the CoV values among the different lesion categories as well as between SUVmax and SUVmean values. Furthermore, Pearson’s correlation analysis showed that SUVmax (r = 0.7577, *p* < 0.0001) and SUVmean (r = 0.5722, *p* < 0.0001) were directly and significantly correlated to CoV values.

In addition, volumetric parameters such as MTV and TLG were calculated on all lesions of each patient for a total of 419 lesions including 84 primary tumor lesions, 163 lymph nodes and 172 distant metastases. Mean MTV and TLG values in the 84 primary tumors were 66.79 ± 10.74 mL and 382.77 ± 56.83 g, respectively. Moreover, MTV_TOT_ and TLG_WB_ that reflect whole-body tumor burden were calculated by summing all measurable lesions detected in each patient. Mean MTV_TOT_ and TLG_WB_ values were 140.85 ± 16.97 mL and 756.24 ± 88.60 g, respectively.

After a mean follow-up period of 11 months, 53 patients had progressive disease and died, 16 had progression and were alive whereas 15 patients had stable disease. Survival analysis was then performed including age, gender, histology, stage, imaging parameters derived from primary tumors (diameter, SUVmax, SUVmean, CoV, MTV and TLG) and whole-body volumetric parameters (MTV_TOT_ and TLG_WB_). SUVmax, SUVmean and CoV of primary tumors were dichotomized using the median value as threshold (11.63, 5.05 and 0.38, respectively). Table 3 reports the results of univariate analysis for both OS and PFS. OS was predicted by CoV (*p* = 0.0184), MTV_TOT_ (*p* = 0.0050), TLG_WB_ (*p* = 0.0108) and stage (*p* = 0.0041).

**Figure 2:**
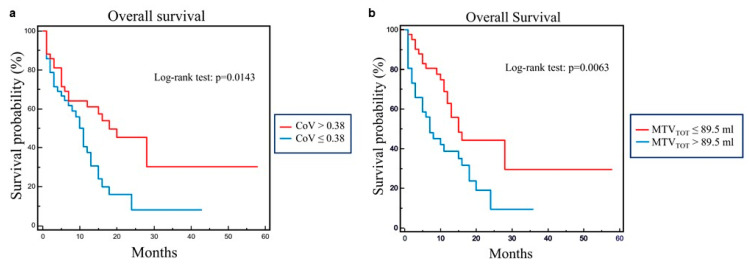
Overall survival by Kaplan–Meier analysis and log-rank test in 84 patients with advanced NSCLC using CoV (**a**) and MTV_TOT_ (**b**) median value as cutoff. Significant different OS between patients with CoV higher and lower than 0.38 (χ^2^ = 6.0005, *p* = 0.0143) (**a**). Significant different OS between patients with MTV_TOT_ lower and higher than 89.5 mL (χ^2^ = 7.4546, *p* = 0.0063) (**b**).

These variables along with age were tested in multivariate analysis and age, CoV, MTV_TOT_ and stage were retained in the model (χ^2^ = 24.4730, *p* = 0.0001). Subsequently, Kaplan–Meier analysis and long-rank testing were performed using the median values of CoV (0.38) and MTV_TOT_ (89.5 mL) as cutoff showing that patients with CoV > 0.38 had significantly better OS as compared to those with CoV ≤ 0.38 (χ ^2^= 6.0005, *p* = 0.0143) (Figure 2a). Moreover, OS was significantly better in patients with MTV_TOT_ ≤ 89.5 mL than those with MTV_TOT_ > 89.5 mL (χ^2^ = 7.4546, *p* = 0.0063) (Figure 2b).

**Figure 3:**
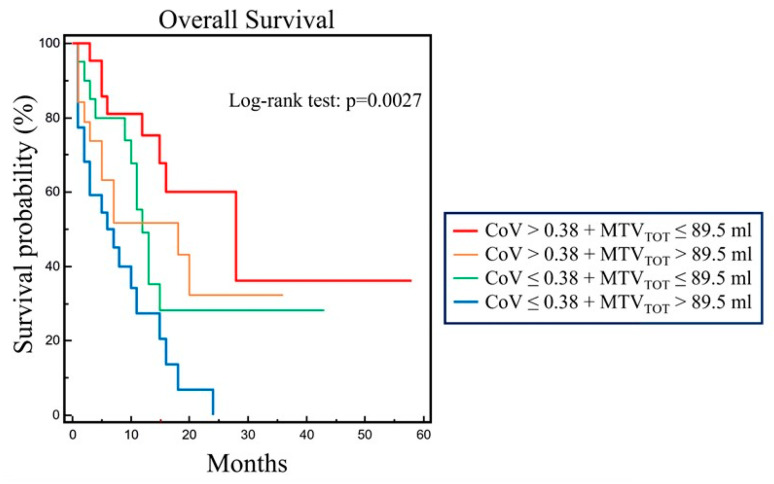
Overall survival by Kaplan-Meier analysis and log-rank test in 84 patients with advanced NSCLC combining CoV and MTV_TOT_ respective median values as cutoff. The four survival curves obtained showed statistically significant difference (χ^2^ = 14.1719, *p* = 0.0027). In particular, patients with COV ≤ 0.38 and MTV_TOT_ > 89.5 mL had the worst OS whereas patients with CoV > 0.38 and MTV_TOT_ ≤ 89.5 mL showed the best OS.

Finally, CoV and MTV_TOT_ were tested in the four possible combinations by using the respective median value as cut off for Kaplan–Meyer analysis. A statistically significant difference among the four survival curves was found (χ^2^ = 14.1719, *p* = 0.0027). In fact, patients with COV ≤ 0.38 and MTV_TOT_ > 89.5 mL had the worst prognosis, while the best OS was observed in patients with COV > 0.38 and MTV_TOT_ ≤ 89.5 mL. Moreover, the other two subgroups had an intermediate pattern of survival (Figure 3).

At univariate analysis, PFS was significantly predicted by MTV_TOT_ (*p* = 0.0046), TLG_WB_ (*p* = 0.0056), and stage (*p* = 0.0039); these variables along with age were tested in multivariate analysis and only MTV_TOT_ and stage were retained in the model (χ^2^ = 14.6020, *p* = 0.0007). By Kaplan–Meyer analysis and long-rank test patients with MTV_TOT_ ≤ 89.5 mL showed a significantly prolonged PFS as compared to those with MTV_TOT_ > 89.5 mL (χ^2^ = 9.2252, *p* = 0.0024).

## 4. Discussion

The present study shows that the first-order parameter CoV and the whole-body volumetric parameter MTV_TOT_ derived from ^18^F-FDG PET/CT may both predict the clinical outcome of patients with advanced NSCLC. In particular, patients with CoV of primary tumors lower than the threshold had worse OS suggesting that a high expression of the glycolytic phenotype in a large proportion of tumor cells, producing a small SD and a high SUVmean, can be associated with aggressive disease, poor response to treatment and consequent poor prognosis. On the contrary, patients with CoV higher than the threshold may have tumors with a low proportion of cells with a glycolytic phenotype that would lead to less aggressive disease, better response to therapy and improved survival. Moreover, also patients with MTV_TOT_ higher than the threshold had worse outcomes and increased risk of progression due to their high tumor burden. Despite tumor heterogeneity of NSCLC occurring at both genetic and molecular levels, the glycolytic phenotype is retained by primary tumors, lymph node metastases and distant metastases with no statistically significant variations of CoV. Therefore, the glycolytic phenotype at different tumor sites has similar characteristics showing a comparable degree of heterogeneity. A further consideration is that the large panel of driver mutations found in NSCLC can modulate in a similar manner the glycolytic phenotype.

However, the limitations of our study including the retrospective design, the relatively limited number of patients and heterogeneous histology may require validation of the results in a larger prospective study. In fact, the use of stringent criteria for the prospective enrollment of a large number of patients may reduce the heterogeneity caused by different histology of lung lesions avoiding any potential variability in the study population. In addition, although the interobserver variability in our study was limited by the fact that the regions of interest were drawn using an automated contouring program, different segmentation methods and thresholds may be compared to further reduce the variation in the extraction of texture features.

Tissue biopsy or random sampling cannot encompass the full extent of phenotypic or genetic variation within a tumor and it cannot be used as a representative parameter of intratumoral heterogeneity across the entire tumor volume. Therefore, it would be helpful to use non-invasive methods to assess tumor heterogeneity for survival prediction and selection of patients who may need more intensive therapeutic regimens. Texture analysis is emerging as a powerful tool with an increasing number of published studies for a quantitative assessment of tumor heterogeneity by analyzing the distribution and relationship of pixel or voxel grey levels in the image [22,23]. In particular, the heterogeneity of FDG uptake in primary lung tumors was evaluated by taking into account a number of texture parameters sometimes combined in statistical models [24,25]. Lovinfosse et al. [26] studied 63 NSCLC patients in stage I that were subjected to ^18^F-FDG PET/CT scan and then treated by stereotactic body radiation therapy. Dissimilarity, a second-order feature of texture analysis that describes the local variation of the grey level of voxel pairs in an image, was found to be a strong and independent predictor of OS since the higher the dissimilarity the better the OS. Moreover, survival analysis by the Kaplan–Meier method showed that patients with dissimilarity lower than or equal to the cutoff level had a higher risk of recurrence as compared to patients having dissimilarity higher than the threshold. Therefore, despite the more sophisticated calculation of dissimilarity, the behavior of this parameter is in agreement with the findings obtained with CoV. Similarly, coarseness, a higher-order texture feature that indicates the grey level difference between a central voxel and its neighborhood, was evaluated in lung cancer patients candidate for chemoradiotherapy and subjected to ^18^F-FDG PET/CT before treatment [13]. In this study, a high coarseness, i.e., a relatively uniform grey level in a ROI drawn around a primary lung tumor [23], was associated with an increased risk of progression and death. These findings were again in agreement with the behavior of CoV since a low CoV value is indicative of a higher homogeneity of glycolytic phenotype. Furthermore, significantly greater pre-treatment COV values were found in patients with locally advanced NSCLC who responded to treatment compared with non-responders [27]. In another study, a higher CoV value of primary NSCLC in newly diagnosed patients with clinically suspected N2 predicted the presence of lymph node metastases at histopathological examination [28]. The latter results are apparently in contrast with our findings since a high CoV in our study is associated with longer survival. This apparent discrepancy can be explained by the fact that CoV is directly correlated with SUVmax and SUVmean and both are indices of tumor aggressiveness. Considering other types of cancer, high CoV values were correlated with a longer PFS in patients with locally advanced rectal cancer [29].

In addition to its prognostic value, CoV has been used also to discriminate metastatic and normal regional lymph nodes in NSCLC patients. In fact, significantly higher CoV values were found in involved lymph nodes as compared to normal lymph nodes and these observations may be ascribed again to its correlation with SUVmax and SUVmean [30].

Texture analysis by generating a large set of data-driven information often lacks biological correlates and radiomic features can be predictive of a good or poor prognosis without a real understanding of their biological meaning. In addition, the biological comprehension of a set of radiomic features may vary depending on the tracer used. In the case of ^18^F-FDG, the radiomic features reflect the local and regional heterogeneity of the glycolytic phenotype. When analyzing the uptake of a radioligand, such as a ^68^Ga-labeled somatostatin analog, these features reflect the heterogeneity of receptor expression [31] and the higher its heterogeneity the worst the response to receptor targeted therapy. Similarly, if texture analysis is focused on the expression of a differentiation marker in a tumor, the higher local and regional variation of its expression can be associated with more aggressive disease and the worst prognosis [32].

Several attempts have been performed to find the relationship between a radiomic signature and clinical findings [33], genomic profiles [34,35,36], or pathological correlates [37,38] and, although these studies provided many biological clues for the interpretation of radiomic features, evidence of their association with specific molecular processes and pathways remains elusive. At present, a high expectation relies upon the analysis of single-cell genomics, proteomics and transcriptomics of tumor samples that had been subjected to radiomic analysis. The biological validation of radiomic features in these studies can lead to widespread use of these methods based on a higher comprehension of their meaning [39].

## 5. Conclusions

Our study shows that the coefficient of variation is an independent prognostic factor for predicting survival in NSCLC patients. This simple first-order parameter can be easily interpreted thus providing information on the variability of the glycolytic phenotype in primary and metastatic lesions. CoV’s biological meaning is different but equally important as compared to that of MTV_TOT_ which represents the whole tumor burden. Therefore, the combination of both parameters may improve the risk stratification of NSCLC patients allowing them to receive more personalized therapeutic approaches.

## Figures and Tables

**Table 1:** Clinical characteristics, histology, stage and treatment of 84 patients with advanced NSCLC.

Characteristic	*N*°	%
**Patients**	84	
**Age**		
Mean ± SD	66 ± 12	
Range	38–87	
**Gender**		
Male	59	70
Female	25	30
**Histology**		
Adenocarcinoma	41	49
Squamous cell carcinoma	20	24
Large cell carcinoma	3	3
Not otherwise specified	20	24
**TNM stage**		
IIIA	7	8
IIIB	11	13
IIIC	9	11
IVA	20	24
IVB	37	44
**Treatment**		
Chemotherapy	50	60
Chemoradiotherapy	4	4
Chemotherapy/Immunotherapy	15	18
No cancer therapy	15	18

SD, Standard Deviation.

**Table 2:** Volume-based imaging parameters determined by ^18^F-FDG PET/CT and expressed as mean ± SD and median value.

Lesions	*N*°	SUVmax		SUVmean		CoV	
		Mean ± SD	Median	Mean ± SD	Median	Mean ± SD	Median
Primary tumors	84	12.17 ± 5.86	11.63	5.44 ± 2.04	5.05	0.36 ± 0.13	0.38
Regional nodes	48	10.97 ± 6.96	10.29	4.67 ± 1.85	4.35	0.36 ± 0.14	0.36
Extraregional nodes	17	14.22 ± 10.41	11.08	5.40 ± 2.11	5.14	0.42 ± 0.18	0.41
Liver metastases	9	9.90 ± 4.67	9.41	5.12 ± 1.48	5.50	0.30 ± 0.14	0.23
Bone lesions	23	10.68 ± 5.21	9.54	4.46 ± 1.00	4.35	0.37 ± 0.17	0.35
Other distant metastases	13	10.36 ± 3.82	10.57	4.79 ± 1.19	5.16	0.34 ± 0.13	0.38

CoV, Coefficient of Variation; SD, Standard Deviation.

**Table 3:** Predictors of overall and progression-free survival by univariate analysis of clinical and imaging variables.

Variable	Overall Survival		Progression-Free Survival	
	χ^2^	*p*	χ^2^	*p*
Age	1.2300	0.2673	0.0544	0.8155
Gender	0.3720	0.5418	1.7760	0.1826
Primary tumor diameter	0.0062	0.9374	0.0281	0.8668
Histology	1.6550	0.1982	2.0280	0.1545
SUVmax (≤11.63 vs. >11.63)	0.0767	0.7818	0.0001	0.9954
SUVmean (≤5.05 vs. >5.05)	1.2460	0.2643	1.1890	0.2755
CoV (≤0.38 vs. >0.38)	5.5600	0.0184	2.3350	0.1265
Primary tumor MTV	0.3550	0.5515	0.7230	0.3951
Primary tumor TLG	0.0918	0.7619	0.2600	0.6099
MTV_TOT_	7.8820	0.0050	8.0390	0.0046
TLG_WB_	6.4920	0.0108	7.6680	0.0056
Stage	8.2530	0.0041	8.3320	0.0039

CoV, Coefficient of Variation; MTV, Metabolic Tumor Volume; TLG, Total Lesion Glycolysis; MTV_TOT_, Total Metabolic Tumor Volume; TLG_WB_, Whole-Body Total Lesion Glycolysis.

## Data Availability

The data presented in this study are available on request from the corresponding author.
